# Elevated levels of extracellular vesicles in progranulin‐deficient mice and FTD‐*GRN* Patients

**DOI:** 10.1002/acn3.51242

**Published:** 2020-11-16

**Authors:** Andrew E. Arrant, Skylar E. Davis, Rachael M. Vollmer, Charles F. Murchison, James A. Mobley, Alissa L. Nana, Salvatore Spina, Lea T. Grinberg, Anna M. Karydas, Bruce L. Miller, William W. Seeley, Erik D. Roberson

**Affiliations:** ^1^ Departments of Neurology and Neurobiology Center for Neurodegeneration and Experimental Therapeutics Alzheimer’s Disease Center Evelyn F. McKnight Brain Institute University of Alabama at Birmingham Birmingham Alabama USA; ^2^ Department of Biostatistics University of Alabama at Birmingham Birmingham Alabama USA; ^3^ Department of Surgery University of Alabama at Birmingham Birmingham Alabama USA; ^4^ Department of Neurology Memory and Aging Center UCSF Weill Institute for Neurosciences University of California, San Francisco San Francisco California USA; ^5^ Department of Pathology University of California, San Francisco San Francisco California USA

**Keywords:** frontotemporal dementia, progranulin, extracellular vesicle, exosome

## Abstract

**Objective:**

The goal of this study was to investigate the effect of progranulin insufficiency on extracellular vesicles (EVs), a heterogeneous population of vesicles that may contribute to progression of neurodegenerative disease. Loss‐of‐function mutations in progranulin (*GRN*) are a major cause of frontotemporal dementia (FTD), and brains from *GRN* carriers with FTD (FTD‐*GRN*) exhibit signs of lysosomal dysfunction. Lysosomal dysfunction may induce compensatory increases in secretion of exosomes, EVs secreted from the endolysosomal system, so we hypothesized that progranulin insufficiency would increase EV levels in the brain.

**Methods:**

We analyzed levels and protein contents of brain EVs from *Grn^–/–^* mice, which model the lysosomal abnormalities of FTD‐*GRN* patients. We then measured brain EVs in FTD‐*GRN* patients. To assess the relationship of EVs with symptomatic disease, we measured plasma EVs in presymptomatic and symptomatic *GRN* mutation carriers.

**Results:**

*Grn^–/–^* mice had elevated brain EV levels and altered EV protein contents relative to wild‐type mice. These changes were age‐dependent, occurring only after the emergence of pathology in *Grn^–/–^* mice. FTD‐*GRN* patients (n = 13) had elevated brain EV levels relative to controls (n = 5). Symptomatic (n = 12), but not presymptomatic (n = 7), *GRN* carriers had elevated plasma EV levels relative to controls (n = 8).

**Interpretation:**

These data show that symptomatic FTD‐*GRN* patients have elevated levels of brain and plasma EVs, and that this effect is modeled in the brain of *Grn^–/–^* mice after the onset of pathology. This increase in EVs could influence FTD disease progression, and provides further support for EVs as potential FTD biomarkers.

## Introduction

Loss‐of‐function mutations in the progranulin gene (*GRN*) are among the most common genetic causes of frontotemporal dementia (FTD), accounting for around 5% of all FTD cases and up to 25% of familial FTD cases.^1^, ^2^, ^3^ Most of these mutations cause progranulin haploinsufficiency, primarily through nonsense‐mediated decay.^1^, ^2^ Progranulin haploinsufficiency is, therefore, thought to drive FTD pathogenesis in *GRN* mutation carriers.

Lysosomal dysfunction may be a mechanism by which progranulin haploinsufficiency causes FTD. Individuals homozygous for *GRN* mutations, resulting in nearly complete progranulin deficiency, develop the lysosomal storage disorder Neuronal Ceroid Lipofuscinosis (NCL).^4^, ^5^, ^6^, ^7^, ^8^ Heterozygous *GRN* carriers typically have less than 50% of normal circulating progranulin levels,^9^, ^10^ and develop FTD. However, brains from these FTD‐*GRN* patients exhibit signs of lysosomal dysfunction such as elevated lipofuscin accumulation,^11^ elevated levels of lysosomal proteins,^12^ and impaired activity of the lysosomal enzymes cathepsin D^11^, ^13^ and β‐glucocerebrosidase.^14^, ^15^, ^16^ These lysosomal deficits impair the autophagy‐lysosomal system in progranulin‐insufficient neurons^13^, ^17^, ^18^, ^19^ and may underlie the impaired phagocytosis and antigen processing of progranulin‐insufficient microglia and macrophages.^18^, ^20^, ^21^, ^22^, ^23^

Lysosomes are a key component of the cellular endolysosomal system, a system of vesicular traffic in which early endosomes formed at the cell membrane may recycle back to the cell membrane or mature into late endosomes, which may fuse with lysosomes for degradation of their contents. Lysosomal dysfunction may disrupt endolysosomal trafficking, particularly in multivesicular bodies (MVBs), late endosomal vesicles that contain many intraluminal vesicles. MVBs may fuse with lysosomes for degradation of their contents, but may also undergo exocytosis and release their intraluminal vesicles as exosomes, a type of extracellular vesicle (EV).^24^ Impairment of lysosomal activity results in increased secretion of EVs, possibly as a compensatory response.^25^ In the context of disease, EV secretion may allow cells to reduce their burden of lysosomal storage material^26^ or pathological proteins.^27^, ^28^, ^29^, ^30^ While this may be beneficial for the cells secreting EVs, it may also spread pathological proteins to other cells throughout the brain.^31^, ^32^, ^33^, ^34^, ^35^, ^36^

Data on the effects of progranulin insufficiency on EV secretion are mixed, and may indicate cell‐type–specific changes in EV secretion due to progranulin insufficiency. Fibroblasts from *GRN* mutation carriers secreted fewer EVs than controls, but knockdown of progranulin in SH‐SY5Y cells appeared to increase EV secretion.^37^ There is increasing evidence that EV secretion in the brain plays a role in progression of neurodegenerative disease,^30^, ^33^, ^35^, ^38^, ^39^ so we were particularly interested in how progranulin insufficiency affects EV secretion in the brain. Given the lysosomal abnormalities in the brain of both progranulin‐insufficient mice and FTD‐*GRN* patients, we hypothesized that progranulin insufficiency would increase brain exosome secretion, resulting in an increase in total EV levels in the brain.

Methods have been developed to isolate vesicles from brain tissue that have the physical characteristics and classic protein and nucleic acid markers of EVs such as exosomes,^40^, ^41^ so we tested our hypothesis by analyzing EV levels in brains of progranulin‐insufficient mice and FTD‐*GRN* patients. We also measured EV levels in plasma from *GRN* mutation carriers. As in the brain, we hypothesized that progranulin insufficiency might increase plasma EV levels through either lysosomal dysfunction or increased inflammation. Lymphoblasts from *GRN* carriers accumulate lysosomal storage material, a sign of lysosomal dysfunction.^11^ Additionally, progranulin is highly expressed by monocytes and macrophages, and progranulin‐insufficient macrophages exhibit abnormal cytokine secretion and impaired antigen processing.^20^, ^23^ This approach allowed us to examine EV levels in *GRN* mutation carriers prior to end‐stage disease, as we measured EV levels in plasma from FTD‐*GRN* patients and presymptomatic *GRN* mutation carriers.

## Materials and Methods

### Animals

Wild‐type, *Grn^+/–^,* and *Grn^–/–^* littermates were used for this study, and were generated as previously described.^42^, ^43^ Both male and female mice were included in the study. The mice were housed in an Association for Assessment and Accreditation of Laboratory Animal Care‐accredited facility, under conditions previously described.^44^ All experiments were approved by the Institutional Animal Care and Use Committee of the University of Alabama at Birmingham.

For hemibrain collection, mice were anesthetized with pentobarbital (200 mg/kg Fatal Plus, Vortech Pharmaceuticals) and transcardially perfused with 0.9% saline. Brains were removed, bisected into hemibrains, and immediately frozen on dry ice. Frozen hemibrains were kept at −80°C until EV isolation.

### Patient brain samples

Post mortem brain samples were provided by the University of California, San Francisco (UCSF) Neurodegenerative Disease Brain Bank, plus one case from the University of Alabama at Birmingham (UAB) Alzheimer’s Disease Center. Brains were donated with the consent of the patients or their surrogates in accordance with the Declaration of Helsinki, and the research was approved by the UCSF Committee on Human Research and UAB Institutional Review Board. Tissue blocks were dissected from the inferior frontal gyrus of 5 controls and 13 patients with FTD‐*GRN*. All patients with FTD‐*GRN* carried a pathogenic variant in *GRN* and had FTLD‐TDP, Type A identified at autopsy (Table 1). Clinical and neuropathological diagnoses were made using standard diagnostic criteria.^45^, ^46^, ^47^, ^48^, ^49^

**Table 1:** Patient data for brain samples.

Case	Group	Sex	Age at death	PMI (hours)	Clinical Diagnosis	Primary Path Diagnosis*	Other Path Diagnosis**
1	Ctrl	F	86	6.4	Control	N/A	AGD, limbic; VBI
2	Ctrl	F	81	30.3	MCI, amnestic	PART	None
3	Ctrl	M	76	8.2	Control	N/A	AGD, limbic
4	Ctrl	M	77	4.9	MCI, executive	AGD	VBI, microinfarct in cerebellar folia
5	Ctrl	F	86	7.8	Control	N/A	VBI, microinfarcts in claustrum and angular gyrus; AGD
6	*GRN*	M	66	10.1	DLB, ?bvFTD	LBD	Incipient FTLD‐TDP‐A
7	*GRN*	M	72	23.8	PPA‐mixed	FTLD‐TDP‐A	Subdural hematoma
8	*GRN*	M	74	30.9	nfvPPA, CBS	FTLD‐TDP‐A	None
9	*GRN*	F	73	20.7	nfvPPA, CBS	FTLD‐TDP‐A	None
10	*GRN*	F	66	7.4	bvFTD	FTLD‐TDP‐A	None
11	*GRN*	M	64	7.2	bvFTD	FTLD‐TDP‐A	None
12	*GRN*	F	70	9.1	PPA, unspecified	FTLD‐TDP‐A	Hippocampal Sclerosis, VBI, ischemic infarct
13	*GRN*	F	56	7.6	bvFTD	FTLD‐TDP‐A	None
14	*GRN*	F	78	19	mixed FTD	FTLD‐TDP‐A	None
15	*GRN*	F	66	17.1	bvFTD	FTLD‐TDP‐A	None
16	*GRN*	F	64	10.5	CBS	FTLD‐TDP‐A	None
17	*GRN*	M	72	7.2	AD	AD, FTLD‐TDP‐A?	Hippocampal Sclerosis
18	*GRN*	F	69	11	bvFTD	FTLD‐TDP‐A	Pre‐Hippocampal Sclerosis

AGD, argyrophilic grain disease; CAA, Cerebral amyloid angiopathy; CBS, Corticobasal syndrome; DLB, dementia with Lewy bodies; LBD, Lewy body disease; MCI, Mild cognitive impairment; nfv, nonfluent variant; PART, Primary age‐related tauopathy; PMI, post mortem interval, PPA, Primary progressive aphasia; VBI, Vascular brain injury.

* Disease considered most likely to explain the clinical syndrome.

** No control subject had limbic TDP‐43 proteinopathy.

### Patient plasma samples

EVs were isolated from archived plasma samples collected at the University of California, San Francisco. Venous blood was collected in EDTA tubes with the consent of the patients or their surrogates in accordance with the Declaration of Helsinki, and the research was approved by the UCSF Committee on Human Research. Blood samples were centrifuged at 1500 x *g* for 15 minutes to isolate plasma, which was stored at −80°C until analysis. Samples were analyzed from 8 controls, 7 presymptomatic *GRN* carriers, and 12 symptomatic *GRN* patients. Symptomatic *GRN* patients were defined as individuals carrying a pathogenic *GRN* mutation who were also diagnosed with a neurodegenerative disorder. While most *GRN* patients were diagnosed with an FTD spectrum disorder, several presented with clinical syndromes consistent with amnestic MCI or Alzheimer’s disease (cases 11, 14, 18) or Parkinson’s disease (case 12). Presymptomatic *GRN* carriers carried a pathogenic *GRN* mutation, but were clinically normal at the time of sample collection. Two patients (cases 23 and 27) developed symptoms at later times, which is noted in Table 2.

**Table 2:** Patient data for plasma samples.

Case	Group	Sex	Age at Collection	Age at Symptoms Onset	Clinical Diagnosis
1	Ctrl	F	82	0	–
2	Ctrl	F	69	0	–
3	Ctrl	F	78	0	–
4	Ctrl	M	72	0	–
5	Ctrl	F	74	0	–
6	Ctrl	F	59	0	–
7	Ctrl	F	61	0	–
8	Ctrl	F	56	0	–
9	*GRN*	F	75	72	FTD^a^
10	*GRN*	F	55	51	FTD^a^
11	*GRN*	M	70	62	Alzheimer’s Disease (probable)^c^
12	*GRN*	M	62	**	Parkinson's Disease, cognitively normal
13	*GRN*	F	72	68	nfvPPA
14	*GRN*	F	70	60	MCI
15	*GRN*	F	65	61	FTD^a^
16	*GRN*	F	66	60	FTD^a^
17	*GRN*	M	54	52	bvFTD^b^
18	*GRN*	M	72	67	Alzheimer’s Disease (probable)^c^
19	*GRN*	F	61	54	FTD^a^
20	*GRN*	F	81	81	bvFTD^b^/PSP
21	presym‐*GRN*	M	61	–	–
22	presym‐*GRN*	F	56	–	Mild Behavioral Issues
23	presym‐*GRN*	M	58	59	MCI, amnestic*
24	presym‐*GRN*	F	56	–	–
25	presym‐*GRN*	M	43	–	–
26	presym‐*GRN*	M	62	–	–
27	presym‐*GRN*	F	70	70	Subjective Cognitive Impairment*

bvFTD, behavioral variant frontotemporal dementia; MCI, mild cognitive impairment; nfvPPA, nonfluent variant primary progressive aphasia.

^a^ Diagnosed according to the Neary criteria.^47^

^b^ Diagnosed according to the FTD Consortium criteria.^48^

^c^ Diagnosed according to the NINCDS criteria^50^.

* Clinically normal at time of sample collection.

** Patient had a prior diagnosis of Parkinson’s disease, but had not developed cognitive symptoms.

### Brain EV isolation

**Figure 1:**
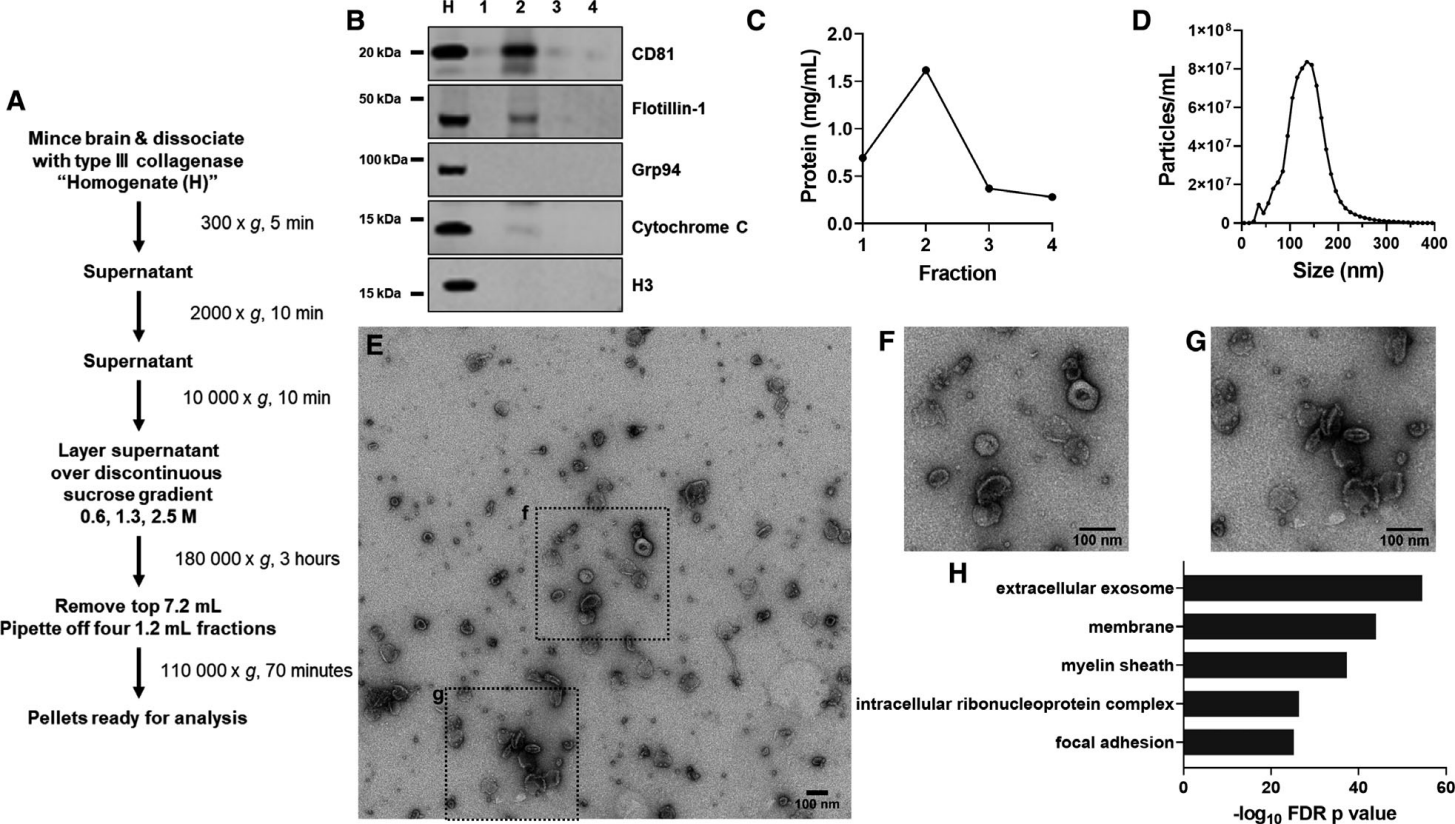
Preliminary Analysis of Mouse Brain EVs. A, EVs were isolated from frozen mouse hemibrains using a method adapted from that of Vella, et al.^41^ After centrifugation over a sucrose gradient, four fractions were harvested. B, The second fraction was enriched for EV markers such as CD81 and flotillin‐1, with depletion of markers for endoplasmic reticulum/Golgi apparatus (Grp94), mitochondria (cytochrome C), and nuclei (H3). For this analysis, the brain homogenate (H) was used as a control. C, Fraction 2 was also enriched for total protein content. Fraction 2 contained vesicles in the size range of exosomes and microvesicles (D), with a peak at around 135 nm in preliminary analysis. E–G, These vesicles exhibited typical EV morphology under transmission electron microscopy, and, H, proteomic analysis of these vesicles revealed strong enrichment for exosomal proteins (top 5 (lowest *P* value) GO Cellular Component terms, DAVID Bioinformatics Resources 6.8). Representative EVs are shown in E–G with 100 nm scale bars. F and G are enlarged portions of E as outlined with dashed lines.

Brain EV were isolated using a method adapted from Vella, et al^41^ (Figure 1A). Frozen hemibrains were weighed prior to slicing into 1–2 mm slices. The slices were then shaken at 225 rpm for 15 minutes at 37°C in Hibernate A medium (Life Technologies) containing 75 units of Type 3 collagenase (Worthington) per mL. Eight‐hundred μL of this collagenase solution were added per 100‐mg tissue. The slices were then triturated three times with a 25‐mL serological pipette, then returned to the incubator for another 5 minutes with shaking. A protease inhibitor cocktail (Halt protease inhibitor cocktail, ThermoFisher Scientific) was added to the solution to a 1X concentration, and the slices were triturated three times with a 1‐mL pipette. This solution is referred to as the homogenate throughout this manuscript.

The homogenate was then subjected to differential centrifugation to isolate brain EVs (Figure 1A). All centrifugation was performed at 4°C. An initial spin at 300 x *g* for 5 minutes pulled down large pieces of tissue and whole cells. This pellet was resuspended in lysis buffer (50 mM Tris, 150 mM NaCl, 5 mM EDTA, 1% Triton X‐100, 0.1% sodium deoxycholate) and is referred to as “P1”. The supernatant “S1” was then spun at 2000× *g* for 10 minutes, and the resulting supernatant was spun at 10 000× *g* for 30 minutes. This supernatant was spun for 3 hours at 180 000× *g* over a discontinuous sucrose gradient of 0.6, 1.3, and 2.5 M sucrose. After centrifugation, the top 7.2 mL of liquid was removed and discarded. The remaining liquid was removed in 1.2 mL increments (fractions 1–4). These fractions were then brought to 4 mL with PBS and centrifuged at 110 000× *g* for 70 minutes. The pellets from each fraction were resuspended in 40‐μL TBS and frozen at −80°C until further analysis.

Consistent with prior reports,^41^ EVs were concentrated in the second fraction of the gradient, which was highly enriched for EV markers (Figure 1B) and contained higher levels of total protein than the other fractions (Figure 1C). Fraction 2 had low or absent levels of markers for other organelles such as the endoplasmic reticulum/Golgi apparatus (Grp94), mitochondria (cytochrome C), and nuclei (histone H3) (Figure 1B). Fraction 2 contained vesicles of the typical size range of EVs such as exosomes and microvesicles (Figure 1D) that displayed typical EV morphology under transmission electron microscopy (Figure 1E–G). Proteomic analysis of fraction 2 confirmed enrichment for EV proteins (“extracellular exosome”, Figure 1H). Our subsequent analyses of brain EVs, therefore, focused on fraction 2 prepared under these conditions.

Except for nanoparticle tracking profiles, data were corrected for starting tissue weight prior to analysis. Despite being conducted by an experimenter blind to sample group identity, significant group differences were observed in starting hemibrain weight for 2‐ to 3‐month‐old mice, and in post mortem tissue slices (Figure S1). Three mice were excluded for having hemibrain weights greater than 2 standard deviations below the total group mean (one in the 2‐ to 3‐month‐old group and two in the 12‐ to 13‐month‐old group).

### Plasma EV isolation

EVs were isolated from plasma using the protocol of Thery et al.^51^ A uniform volume of plasma per patient (1.8 mLs) was diluted 1:1 with PBS, then centrifuged as follows, taking the supernatant for each subsequent step: 2000× *g*, 30 minutes; 12 000× *g*, 45 minutes; 110 000× *g*, 2 hrs. The resulting pellet was resuspended in PBS, filtered through a 0.22 μm syringe filter, and then spun at 110 000× *g* for 70 minutes. This pellet was again resuspended in PBS, then spun at 110 000× *g* for 70 minutes. This final pellet was resuspended in TBS and frozen at −80°C.

### Nanoparticle tracking analysis

Nanoparticle tracking analysis was performed with a Nanosight NS300 particle imaging system (Malvern Panalytical). EV samples were diluted 1:500 (mouse) or 1:250 (human) in PBS prior to analysis. Data were collected from each sample over five 1‐minute intervals, which were averaged to give the final result for each sample.

### Electron microscopy

EV samples were diluted with TBS, pipetted onto copper grids, and stained with uranyl acetate. Grids were then imaged on a Technai Spirit T12 transmission electron microscope (ThermoFisher).

### Western blot

Homogenates and fractions 1–4 were analyzed by western blot. Protein concentration was determined by Bradford (ThermoFisher) or BCA (ThermoFisher) assay. Homogenates were loaded at 15 μg of protein per well. Since protein content in the EV fraction is related to EV concentration, EV samples were loaded volumetrically (23 μL of the 40 μL fraction) to accurately represent EV concentration in brain or plasma samples.

Samples were diluted with LDS sample buffer (ThermoFisher) and Bolt sample reducing agent (ThermoFisher) prior to loading on 4–12% bis‐tris polyacrylamide gels (ThermoFisher). After electrophoresis, protein from the gels was transferred to Immobilon‐FL PVDF (MilliporeSigma), and incubated overnight with primary antibody. The following primary antibodies were used: CD81 (sc‐166029, mouse monoclonal, Santa Cruz Biotechnology), CD9 (sc‐13118, mouse monoclonal, Santa Cruz Biotechnology), Flotillin‐1 (610820, mouse monoclonal, BD Transduction Laboratories), HSP‐70 (for brain samples, sc‐32239, mouse monoclonal, Santa Cruz Biotechnology; for plasma samples, ab94368, rabbit polyclonal, Abcam), Grp94 (sc‐32249, mouse monoclonal, Santa Cruz Biotechnology), Cytochrome C (sc‐13156, mouse monoclonal, Santa Cruz Biotechnology), Histone H3 (06‐755, rabbit polyclonal, MilliporeSigma), and GluR4 (23350‐1‐AP, rabbit polyclonal, Proteintech). Blots were probed with species‐matched IRdye‐conjugated secondary antibodies (Li‐COR Biosciences) and scanned on an Odyssey scanner (Li‐COR Biosciences).

### Sample preparation for mass spectrometry analysis

EVs isolated from mouse brain were lysed in 1x final NuPAGE LDS Sample Buffer (ThermoFisher) via sonication in an ultrasonic water bath for 20 minutes. Following protein quantitation using an EZQ Protein Quantitation Kit (ThermoFisher), 5 µg of protein per sample was separated onto a NuPAGE 10% Bis‐Tris Protein gel (ThermoFisher) at 200V constant for 15min. The gel was stained using a Colloidal Blue Staining Kit (ThermoFisher) following the manufacturer’s instructions. Each gel lane was excised (three equal‐sized fractions per sample) and digested overnight at 37°C with Pierce Trypsin Protease, MS Grade (Thermo Scientific), as per manufacturer’s instruction. Digests were reconstituted in 0.1% FA in 5:95 ACN:ddH2O at ~0.1 µg/uL.

### nLC‐ESI‐MS2 analysis & database searches

Peptide digests (8 µL each) were injected onto a 1260 Infinity nHPLC stack (Agilent Technologies), and separated using a 100 micron I.D. × 13.5 cm pulled tip C‐18 column (Jupiter C‐18 300 Å, 5 micron, Phenomenex). This system runs in‐line with a Thermo Orbitrap Velos Pro hybrid mass spectrometer, equipped with a nano‐electrospray source (ThermoFisher), and all data were collected in CID mode. The nHPLC was configured with binary mobile phases that included solvent A (0.1% FA in ddH2O) and solvent B (0.1% FA in 15% ddH2O/ 85% ACN), programmed as follows: 10 minutes @ 5% B (2µL/ min, load), 90 minutes @ 5%‐40% B (linear: 0.5 nL/ min, analyze), 5 minutes @ 70% B (2µL/ min, wash), 10 minutes @ 0%B (2µL/ min, equilibrate). Following each parent ion scan (300‐1200m/z @ 60k resolution), fragmentation data (MS2) were collected on the top 15 most intense ions. For data‐dependent scans, charge state screening and dynamic exclusion were enabled with a repeat count of 2, repeat duration of 30 seconds, and exclusion duration of 90 seconds.

The XCalibur RAW files were collected in profile mode, centroided and converted into MzXML using ReAdW v. 3.5.1. The data were searched using SEQUEST, which was set for two maximum missed cleavages, a precursor mass window of 20 ppm, trypsin digestion, variable modification C @ 57.0293, and M @ 15.9949. Searches were performed with a species‐specific subset of the UniRef100 database.

### Peptide filtering, grouping, and quantification

The list of peptide IDs generated based on SEQUEST (ThermoFisher) search results was filtered using Scaffold (Protein Sciences). Scaffold filters and groups all peptides to generate and retain only high confidence IDs while also generating normalized spectral counts (N‐SC’s) across all samples for the purpose of relative quantification. The filter cut‐off values were set with minimum peptide length of >5 AA’s, with no MH + 1 charge states, with peptide probabilities of >80% C.I., and with the number of peptides per protein ≥2. The protein probabilities were then set to a >99.0% C.I., and an FDR <1.0. Scaffold incorporates the two most common methods for statistical validation of large proteome datasets, the false discovery rate (FDR), and protein probability.^52^, ^53^, ^54^ Relative quantification across experiments were then performed via spectral counting,^55^, ^56^ and when relevant, spectral count abundances were then normalized between samples.^57^ Normalized spectra and unique peptide counts are shown in Table S1.

### Experimental design & statistics

EV isolation and analysis was performed by experimenters blinded to experimental group. Nanoparticle tracking profiles (concentration by particle size) were analyzed by repeated measures ANOVA with factors of particle size and genotype (or patient group) using GraphPad Prism 8. For mouse brain samples, 50–150 nm particle concentration, fraction 2 protein content, and western blot data were analyzed by ANOVA (factor of genotype). Nanoparticle tracking profiles for patient samples were analyzed by repeated measures ANOVA, and 55–155 nm particles in plasma samples were analyzed by ANOVA (factor of group). Western blot data for patient samples (brain and plasma) were analyzed by nonparametric methods due to very high levels in some patients. Data from brain samples were analyzed by Mann‐Whitney test, and data for plasma samples were analyzed by Kruskal‐Wallis test (factor of group). For ANOVA and Kruskal‐Wallis test, significant main effects or interactions were followed by post hoc testing compared to controls. These analyses were performed with GraphPad Prism 8, with two‐tailed *p* values and α set at 0.05. Except where specified, data are shown as mean ± SEM.

For proteomic data, enrichment analysis was performed using David Bioinformatics Resources 6.8.^58^, ^59^ Protein spectra data were analyzed to compare protein abundance between mice according to age and genotype using negative binomial regression models implemented using Bioconductor in R via the DESeq2 package.^60^ Spectra values were floored with zero count proteins removed from the dataset and relative log2‐fold changes calculated for contrasts of interest among the four groups. One aged *Grn^–/–^* mouse was removed from the analysis after sensitivity assessment indicated that it inappropriately drove certain effects. Abundance thresholds required at least 16x fold‐change to be considered biologically meaningful with a significance threshold of 10^‐7^ to account for multiplicity. Notable protein species were identified and annotated according to accession and mapped using the GO.db gene ontology database.

## Results

### Isolation of EVs from mouse hemibrains

**Figure 2:**
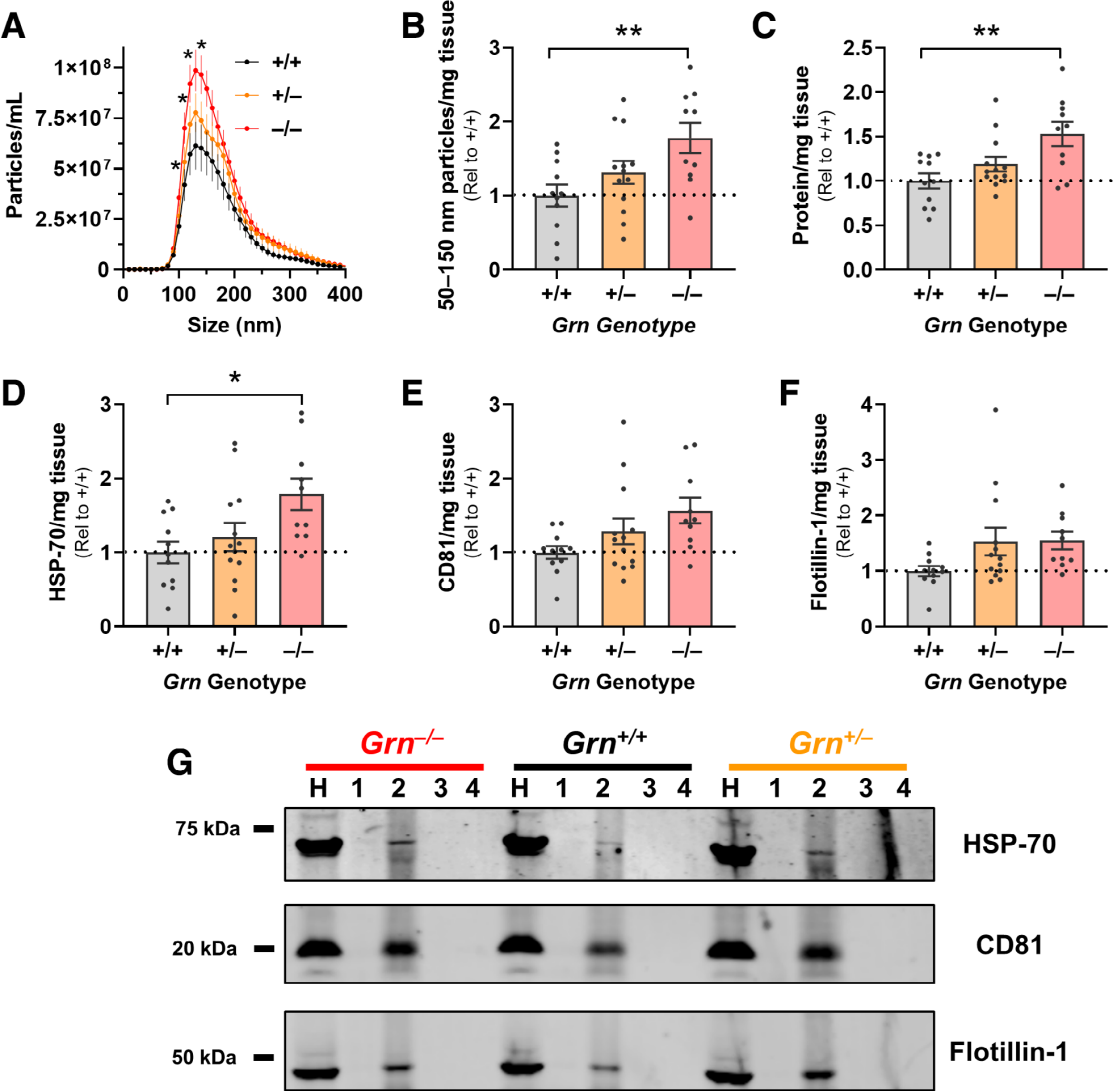
Elevated Levels of Brain EVs in 12‐ to 13‐month‐old *Grn^–/–^* Mice. Brain EVs were isolated from wild‐type, *Grn^+/–^*, and *Grn^–/–^* littermates, and the levels of EVs in fraction 2 were compared using several methods. A, Nanoparticle tracking analysis revealed more vesicles of exosomal size in *Grn^–/–^* mice than wild‐type (RM ANOVA genotype x particle size interaction, *P* < 0.0001, *P < 0.05 by Dunnett’s post hoc test). B, This increase in exosome‐sized vesicles persisted when corrected for hemibrain weight in *Grn^–/–^* mice (ANOVA effect of genotype, *P* = 0.0133, **P = 0.0070 by Dunnett’s post hoc test). C, Fraction 2 from *Grn^–/–^* mice also contained more total protein than wild‐type mice (ANOVA effect of genotype, *P* = 0.0040, **P = 0.0021 by Dunnett’s post hoc test). Finally, fraction 2 from *Grn^–/–^* mice contained significantly more HSP‐70 (D, ANOVA effect of genotype, *P = *0.0206, *P = 0.0138 by Dunnett’s post hoc test) and trended toward having higher levels of CD81 (E, ANOVA effect of genotype, *P* = 0.0562) and flotillin‐1 (F, ANOVA effect of genotype, *P* = 0.0857) than wild‐type. G, The other fractions contained undetectable levels of these proteins. All data are corrected for hemibrain weight except for the nanoparticle tracking profiles in A. n = 10–13 mice per genotype. H = brain homogenate.

To test our hypothesis that progranulin insufficiency would increase EV levels in the brain, we adapted previously reported methods for isolating EVs from frozen brain tissue (Figure 1).^41^ We began by comparing levels of brain EVs in 12‐ to 13‐month‐old wild‐type, *Grn^+/–^*, and *Grn^–/–^* littermates. At this age, *Grn^–/–^* mice exhibit robust lysosomal abnormalities and lipofuscinosis.^43^, ^61^, ^62^, ^63^, ^64^ *Grn^+/–^* mice also exhibit similar, but milder lysosomal abnormalities at around 12 months of age.^65^, ^66^ Nanoparticle tracking analysis of brain EVs from fraction 2 revealed a significantly higher number of EVs in *Grn^–/–^* mice relative to wild‐type (Figure 2A). Since the vesicles isolated from mouse brains likely include multiple EV subtypes, we assessed vesicles of the size range of exosomes (50–150 nm) to determine if the increase in EVs might be associated with endolysosomal dysfunction. EVs of exosomal size were also elevated in *Grn^–/–^* mice relative to wild‐type (Figure 2B). Fraction 2 contained more protein in *Grn^–/–^* mice than in wild‐type (Figure 2C), as well as higher levels of the EV‐enriched protein HSP‐70 (Figure 2D,G). Levels of the EV‐enriched proteins CD81 (Figure 2E,G) and flotillin‐1 (Figure 2F,G) also trended higher in *Grn^–/–^* mice relative to wild‐type littermates.

### Normal levels of brain EVs in 2‐ to 3‐month‐old Grn^–/–^ mice

After observing elevated levels of brain EVs at an age (12–13 months) at which *Grn^–/–^* mice have robust lysosomal abnormalities, we next tested whether *Grn^–/–^* mice have elevated levels of brain EVs before the onset of these abnormalities. We isolated brain EVs from 2‐ to 3‐month‐old wild‐type, *Grn^+/–^*, and *Grn^–/–^* littermates. At this age there are some changes in lysosomal enzyme activity in *Grn^–/–^* brain,^66^ but *Grn^–/–^* mice have not yet developed most of the changes in lysosomal enzyme activity and lipofuscinosis that occur at later ages.^12^, ^61^, ^67^ Consistent with this much milder lysosomal phenotype, we did not observe a significant elevation in brain EVs in 2‐ to 3‐month‐old *Grn^–/–^* mice by nanoparticle tracking analysis (Figure S2A,B), protein assay (Figure S2C), or western blot for EV proteins (Figure S2D‐F) in fraction 2.

### Proteomic analysis of mouse brain EVs

**Figure 3:**
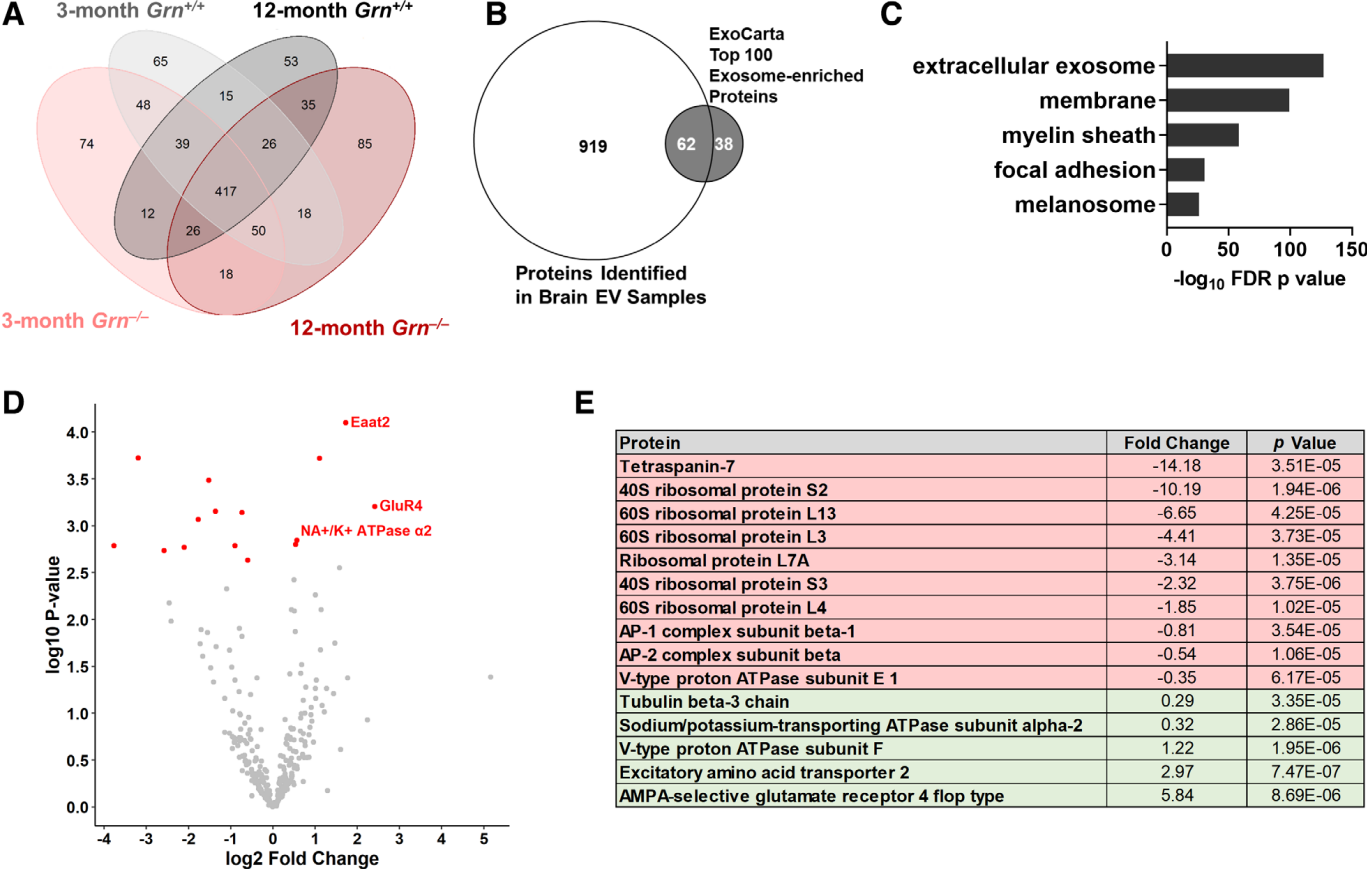
Proteomic Analysis of Brain EVs from Wild‐type and *Grn^–/–^* Mice. Brain EVs (fraction 2) from 2‐ to 3 month‐old and 12‐ to 13 month‐old wild‐type and *Grn^–/–^* mice (n = 4 per group) were analyzed by LC‐MS. A, A total of 981 proteins were detected with a threshold of at least two peptides detected in at least one animal. B, The detected proteins included 62 of the 100 most‐abundant exosomal proteins listed on the ExoCarta database,^68^ and C, enrichment analysis confirmed strong enrichment of exosomal proteins (top 5 (lowest *P* value) GO Cellular Component terms, DAVID Bioinformatics Resources 6.8). Preliminary analysis of the protein spectra revealed genotype differences in proteomic profiles of 12‐ to 13‐month‐old but not 2‐ to 3‐month‐old, wild‐type and *Grn^–/–^* mice. As a result, our subsequent analysis focused on the 12‐ to 13‐month‐old mice. For this analysis, one *Grn^–/–^* mouse was excluded as an outlier based on principal components analysis. D, E, Analysis of the remaining mice revealed 15 proteins with altered levels in *Grn^–/–^* mice.

The age‐dependent increase in the levels of brain EVs in *Grn^–/–^* mice is consistent with our hypothesis that lysosomal dysfunction due to progranulin insufficiency increases EV secretion in *Grn^–/–^* brain. We also hypothesized that lysosomal dysfunction could alter EV contents in *Grn^–/–^* mice. To test this hypothesis, we conducted proteomic analysis of brain EVs from wild‐type and *Grn^–/–^* littermates at ages 2–3 and 12–13 months. A total of 981 proteins were detected (Figure 3A), which included 62 of the 100 most common exosomal proteins listed in the ExoCarta database^68^ (Figure 3B). Enrichment analysis revealed enrichment for exosomal proteins (Figure 3C), as in our preliminary sample.

We compared EV protein contents between groups using the normalized spectra from mass spectrometry. Principal component analysis of these data showed separation based on age (Figure 3D), and an age‐dependent separation between genotypes. Wild‐type and *Grn^–/–^* mice had similar proteomic profiles at 2–3 months of age, but diverged at 12–13 months of age. We, therefore, focused subsequent analysis on brain EVs from 12‐ to 13‐month‐old wild‐type and *Grn^–/–^* mice. For this comparison, we excluded one old *Grn^–/–^* mouse that emerged as an outlier in principal components analysis.

Analysis of 12‐ to 13‐month‐old wild‐type and *Grn^–/–^* mice revealed a total of 15 proteins with altered levels in *Grn^–/–^* mice (Figure 3D,E). Most of the proteins with lower levels in EVs from *Grn^–/–^* mice were ribosomal proteins. Among the proteins increased in EVs from *Grn^–/–^* mice were two proteins highly expressed by astrocytes (Eaat2 and α2 Na+/K + ATPase),^69^ perhaps reflecting the astrocytosis that occurs in 12‐ to 13‐month‐old *Grn^–/–^* mice.^61^ The protein with the largest fold increase in EVs from *Grn^–/–^* mice was GluR4. GluR4 mRNA (*GRIA4*) expression is elevated in FTD‐*GRN* patients, sporadic FTD patients, and a mouse model of FTD due to *CHMP2B* mutations.^70^ We, therefore, immunoblotted for GluR4 in whole‐brain homogenates from a subset of the 12‐ to 13‐month‐old mice used for EV isolation. These blots revealed no significant effect of progranulin genotype on GluR4 levels, although *Grn^–/–^* mice trended toward lower levels of GluR4 in brain homogenates than wild‐type (Figure S3). The increase in EV GluR4, therefore, reflects some process other than an increase in total brain GluR4.

### Elevated levels of EVs in frontal cortex of FTD‐GRN patients

**Figure 4:**
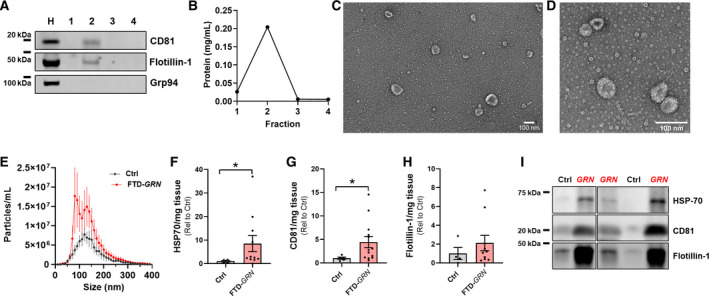
Elevated Levels of EVs in Frontal Cortex of Patients with FTD‐*GRN*. EVs were isolated from frozen post mortem samples of inferior frontal gyrus from controls (n = 5) and patients with FTD‐*GRN* (n = 13) as shown in Figure 1A. A, B, As with mouse brain samples, fraction 2 was enriched for EV marker proteins and total protein content. C, D, Fraction 2 from post mortem samples contained vesicles of typical EV morphology under transmission electron microscopy. E, Nanoparticle tracking analysis revealed vesicles of the size for exosomes and microvesicles, although there was not an overall difference in vesicle concentration between FTD‐*GRN* patients and controls (E, RM ANOVA effect of group, *P* = 0.51). However, levels of the EV marker proteins HSP‐70 (F, Mann‐Whitney test, *P* = 0.0396) and CD81 (G, Mann‐Whitney test, *P* = 0.046) and were elevated in fraction 2 from FTD‐*GRN* patients. Representative blots are shown in I. All data are corrected for slice weight except for the nanoparticle tracking profiles in E. Independent images of representative EVs at different magnifications are shown in C and D with 100 nm scale bars.

We next investigated whether progranulin insufficiency increases EV levels in brains from patients with FTD‐*GRN* (Table 1). We isolated EVs from inferior frontal gyrus of controls (n = 5) and patients with FTD‐*GRN* (n = 13). As with mouse brain samples, fraction 2 was highly enriched for EV markers (Figure 4A) and protein content (Figure 4B), and contained vesicles of typical EV morphology (Figure 4C,D) and size (Figure 4E). While vesicle concentration in fraction 2 did not differ between groups (Figure 4E), fraction 2 of FTD‐*GRN* patients contained higher levels of the EV proteins HSP70 (Figure 4F) and CD81 (Figure 4G) than controls. Levels of flotillin‐1 (Figure 4H) trended higher but were more variable and not significantly elevated. Taken together, these data are consistent with increased levels of EVs in the frontal cortex of FTD‐*GRN* patients.

*Plasma EVs Increase in* GRN *Mutation Carriers After Onset of Symptoms.*

Having observed elevated levels of brain EVs in FTD‐*GRN* patients, we next tested whether progranulin insufficiency might alter EV levels in plasma. A major advantage of examining plasma is the ability to study samples from presymptomatic (n = 7), as well as symptomatic.

(n = 12) *GRN* mutation carriers (Table 2). Presymptomatic individuals were clinically normal at the time of plasma collection. Most of the symptomatic *GRN* patients (8/12) were diagnosed with FTD‐spectrum clinical syndromes (Table 2), although four were diagnosed with other disorders (MCI, probable Alzheimer’s disease, or Parkinson’s disease). As these were clinical diagnoses and neuropathology data were not available for most patients, all symptomatic *GRN* carriers were analyzed as a group.

**Figure 5:**
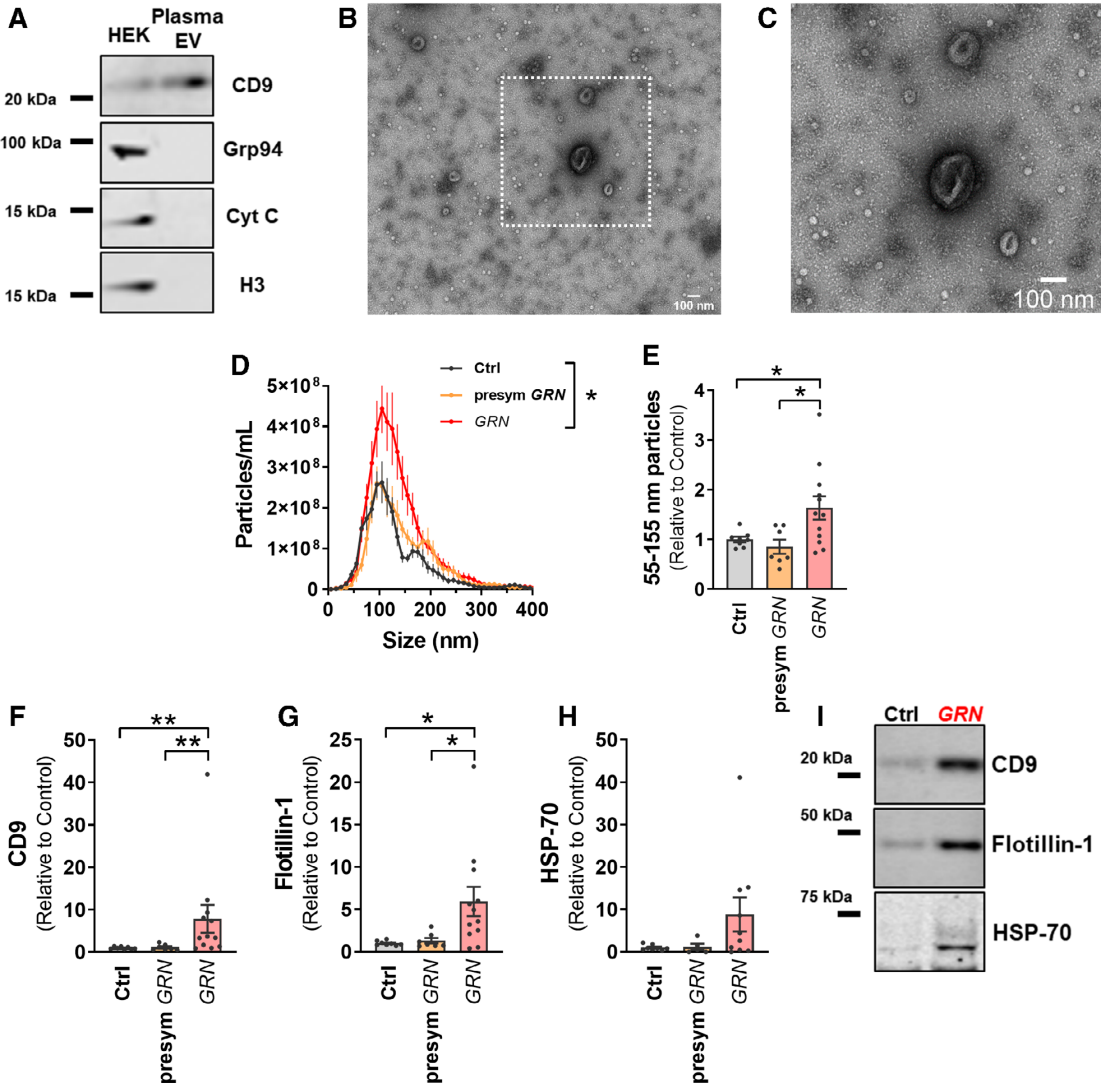
Elevated Levels of Plasma EVs in Patients with FTD‐*GRN*. EVs were isolated by differential centrifugation of frozen plasma samples from controls (n = 8), presymptomatic *GRN* carriers (n = 7), and symptomatic *GRN* patients (n = 12). A, EV pellets contained EV marker proteins, but lacked markers for other organelles (Grp94 – ER/Golgi, cytochrome C – mitochondria, histone H3 – nucleus). Lysate from HEK‐293 cells (HEK) was used as a positive control for organelle markers. B and C Plasma EVs exhibited typical morphology under transmission electron microscopy (scale bars = 100 nm). D, Nanoparticle tracking analysis revealed that plasma from symptomatic *GRN* patients contained significantly more particles of exosomal size than controls and presymptomatic *GRN* carriers (RM ANOVA effect of group, *P* = 0.0447, group x size interaction, *P* < 0.0001, *P = 0.0415 by Dunnett’s post hoc test, E, ANOVA effect of group, *P* = 0.0162, *P < 0.05 by Dunnett’s post hoc test). F–I, Similarly, western blot revealed elevated levels of the EV marker proteins CD9 (F, Kruskal‐Wallis test effect of group, *P* = 0.0021, **P < 0.01 by Dunn’s post hoc test) and flotillin‐1 (G, Kruskal‐Wallis test effect of group, *P* = 0.0182, *P < 0.05 by Dunn’s post hoc test) in symptomatic *GRN* patients compared to both controls and presymptomatic *GRN* carriers. C is an enlarged portion of B, as shown by dashed lines.

EVs were isolated from plasma by differential centrifugation and had the expected protein composition (Figure 5A) and morphology under EM (Figure 5B,C). Similar to results from brain samples (Figure 4E‐I), symptomatic *GRN* patients had higher levels of plasma EVs as assessed by both nanoparticle tracking analysis (Figure 5D,E) and western blot of EV marker proteins (Figure 5F‐I). In contrast, presymptomatic *GRN* carriers did not differ from controls by any of these measures, and had lower levels of exosome‐sized EVs (Figure 5E) and EV marker proteins (Figure 5F,G) than symptomatic *GRN* carriers.

Based on these data, plasma EV levels appear to increase in *GRN* mutation carriers in parallel with the onset of symptomatic neurodegenerative disease. Consistent with this conclusion, the highest levels of CD9 and flotillin‐1 among presymptomatic *GRN* carriers were found in an individual who became symptomatic less than 2 years after plasma collection (Figure S4; Table 2, case 23), although their vesicle concentration by nanoparticle tracking analysis was not among the highest presymptomatic *GRN* carriers. Although these findings are from just one individual, they raise the possibility that plasma EV levels may begin to increase just before the onset of symptoms in *GRN* carriers. (Another *GRN* carrier (Table 2, case 27) who had low plasma EVs developed subjective symptoms within a year after plasma collection, but detailed research examination did not reveal objective evidence of disease onset.)

## Discussion

This study reports increased EV levels in the brain of *Grn^–/–^* mice and both frontal cortex and plasma of FTD‐*GRN* patients, indicating that increased EV secretion is a common effect of progranulin insufficiency. Increased brain EV levels in *Grn^–/–^* mice were found only at ages after the onset of robust lysosomal abnormalities, gliosis, and accumulation of lysosomal storage material.^43^, ^61^, ^62^, ^63^, ^64^, ^67^ Increased EV levels in the frontal cortex of FTD‐*GRN* patients confirm the relevance of this abnormality to FTD. Paralleling the age dependence of brain EV levels in *Grn^–/–^* mice, plasma EVs increased in *GRN* mutation carriers only around the onset of symptomatic neurodegenerative disease, but not in presymptomatic carriers.

We did not observe significant increases in brain EVs in 12‐month‐old *Grn^+/–^* mice, which model the partial progranulin insufficiency of FTD‐*GRN* patients, although they did exhibit nonsignificant trends for increases in several EV measures. While not investigated in this study, *Grn^+/–^* mice might develop elevated levels of brain EVs at older ages, which would be consistent with the age dependence of the increase in brain EVs in *Grn^–/–^* mice, and the milder, later onset changes in lysosomal enzyme abnormalities observed in *Grn^+/–^* mice relative to *Grn^–/–^* mice.^66^ Although *Grn^–/–^* mice model the complete progranulin deficiency that causes NCL in humans, they are a more accurate model than *Grn^+/–^* mice of the endolysosomal dysfunction observed in FTD‐*GRN*.^12^, ^71^ Thus, we consider it likely that similar mechanisms underlie the increased brain EVs of both *Grn^–/–^* mice and FTD‐*GRN* patients.

The disease dependence of the increase in plasma EVs in *GRN* mutation carriers, as well as the age dependence of increased brain EV levels in *Grn^–/–^* mice, indicate that increased EV secretion is likely not a result of progranulin insufficiency *per se*, but rather of cellular/lysosomal dysfunction caused by progranulin insufficiency. This is consistent with a report that fibroblasts from *GRN* mutation carriers secrete fewer EV than controls.^37^ Instead, the increase in EV levels in brains of 12‐month‐old *Grn^–/–^* mice and FTD‐*GRN* patients is likely downstream of other changes associated with the symptomatic stages of disease in humans or the onset of pathology and behavioral deficits in mice. These changes might include buildup of lysosomal storage material and worsening of lysosomal dysfunction, or gliosis and inflammation. Similar mechanisms could drive the increase in plasma EVs in symptomatic *GRN* patients.

As we hypothesized, lysosomal dysfunction could increase EV levels by driving increased exosome secretion. Progranulin insufficiency causes lysosomal dysfunction in neurons,^11^, ^13^, ^15^, ^17^, ^71^ microglia,^21^, ^67^ and macrophages,^72^, ^73^ providing potential cellular sources for increased exosome secretion in both brain and plasma. There is also a temporal correlation between increased EV levels and lysosomal abnormalities in *Grn^–/–^* mice, as the increase in *Grn^–/–^* brain EVs occurs at a roughly similar age as the onset of signs of robust lysosomal dysfunction.^43^, ^61^, ^62^, ^63^, ^64^, ^67^

Inflammation and gliosis could also increase EV levels in brain and plasma of FTD‐*GRN* patients and *Grn^–/–^* mice. Degenerating regions such as the frontal cortex of FTD‐*GRN* patients contain many reactive microglia.^74^ Brains of 12‐month‐old, but not 3‐month‐old *Grn^–/–^* mice have more reactive microglia and astrocytes than wild‐type littermates,^43^, ^61^, ^62^, ^63^ and *Grn^–/–^* mice have exaggerated inflammatory responses to injury.^42^, ^75^, ^76^ *Grn^–/–^* macrophages also exhibit exaggerated inflammatory responses to inflammatory stimuli such as LPS.^18^, ^20^ EVs play a role in various immune functions, including antigen presentation, and some types of immune cells secrete higher levels of EVs when activated.^77^, ^78^, ^79^ Gliosis and inflammation could, thus, potentially increase EV secretion and alter EV protein and microRNA contents.

Further work will be necessary to evaluate the role of EVs in FTD‐*GRN* pathogenesis. EV secretion may have both protective and pathogenic effects in neurodegenerative disease.^30^, ^33^, ^35^, ^38^, ^39^ EV secretion can relieve cells of lysosomal storage material^26^ and pathologic proteins,^27^, ^29^, ^30^ including TDP‐43.^28^ However, EVs can also spread pathologic proteins such as TDP‐43 to nearby cells.^28^, ^32^, ^34^ Due to such spreading, EV secretion contributes to both amyloid^35^ and tau^33^ pathology in mouse models. EVs also contain microRNA, and can spread aberrant microRNA during disease. Abnormal microRNA expression may be involved in FTD pathogenesis,^70^, ^80^ and FTD patients, including FTD‐*GRN*, have altered microRNA in EVs from cerebrospinal fluid.^81^ EVs could, therefore, be a vehicle for aberrant microRNA in FTD‐*GRN*. Finally, EVs from plasma may be a vehicle by which peripheral immune cells could increase inflammation in the brain.^78^ While the net effect of the increase in EV levels remains to be determined, the disease/age dependence of increased brain EV levels suggests that the increase in brain EVs may contribute to progression rather than initiation of FTD in *GRN* mutation carriers.

Further analysis of changes in EV levels and contents during the course of disease may reveal novel biomarkers for FTD‐*GRN*. Prior work has illustrated the potential utility of EVs as FTD biomarkers. Abnormal miRNA has been reported in EVs from cerebrospinal fluid of FTD‐*GRN* patients,^81^ and neural‐derived plasma EVs from sporadic FTD patients have reduced levels of synaptic proteins.^82^ Plasma EVs are particularly attractive as biomarkers with the development of techniques to isolate neural‐^83^, ^84^, ^85^ and astrocyte‐derived^86^, ^87^ EVs from patient plasma. Given the difference in EV levels between presymptomatic and symptomatic *GRN* carriers, longitudinal analysis of plasma EVs from *GRN* carriers might provide insight into cellular changes that occur with onset of symptomatic disease. Analysis of these EVs might provide insight into cellular changes that occur during FTD pathogenesis. For example, the increased astrocytic proteins we observed EVs from *Grn^–/–^* mice (Figure 3D) may reflect the astrocytosis present throughout the brain of *Grn^–/–^* mice.^43^, ^61^, ^62^, ^63^ Additionally, the elevated levels of GluR4 in EVs but not brain homogenates of *Grn^–/–^* mice might reflect a change in neuronal activity or glutamate receptor sorting,^22^, ^88^, ^89^, ^90^ or perhaps secretion by microglia that phagocytized synapses in *Grn^–/–^* mice.^90^

A final question raised by these data is whether EVs levels are also increased in other FTD subtypes. It seems likely that patients with FTD due to *CHMP2B* mutations might have increased brain EV levels. CHMP2B is part of the ESCRT‐III complex that is involved in the formation of intraluminal vesicles that become exosomes after secretion from the cell.^91^ *CHMP2B* mutations disrupt endosome‐lysosome fusion,^92^ and brains from FTD‐*CHMP2B* patients accumulate lysosomal storage material.^93^ Endolysosomal dysfunction may also play a role in pathogenesis of sporadic FTD,^94^ raising the possibility that EV levels may be increased in sporadic FTD. Measurement of EV levels in other FTD subtypes might further illuminate the role of EVs in FTD pathogenesis.

Ongoing refinement of techniques for EV isolation and classification is likely to enable further discovery of how progranulin insufficiency changes brain EVs, which may provide insight into mechanisms of FTD pathogenesis. The EVs we obtained from brain tissue are likely to contain a variety of vesicle subtypes originating from distinct cellular pathways. Our hypothesis that progranulin insufficiency would increase secretion of EVs was largely focused on exosomes, which originate from the cellular endolysosomal pathway. However, current methods of isolation and analysis do not allow for the complete separation of exosomes from other classes of extracellular vesicle.^95^, ^96^ For example, there is some overlap in size and protein markers between exosomes and microvesicles that bud directly from the cell membrane.^96^, ^97^ The typical size range for exosomes is around 50–150 nm.^97^ Microvesicles are larger than exosomes, but smaller microvesicles are of similar size as larger exosomes.^95^ Our nanoparticle tracking profiles reveal the presence of some vesicles larger than 150 nm, supporting the potential presence of microvesicles. An additional consideration is the likely presence of small amounts of cellular debris in our brain EV samples. While our EM analysis revealed that most material from brain tissue had vesicular appearance and was in the appropriate size range for EVs (Figure 1E–G, Figure 4C,D), the presence of some smaller particles was also apparent. Future studies may, therefore, be able to specifically study the various EV subtypes with even less contamination from cellular debris, perhaps revealing even clearer changes in EV secretion due to progranulin insufficiency.

## Conflict of Interest

Dr. Arrant reports grants from the National Institute on Aging and Civitan International Research Center during the conduct of the study. Dr. Grinberg reports grants from AVID Radiopharmaceutics and Eli Lilly, outside the submitted work. Dr. Seeley reports personal fees from Guidepoint Consulting, Corcept Therapeutics, Biogen Idec, and GLG Council, outside the submitted work. Dr. Roberson reports grants from The Bluefield Project to Cure FTD and the National Institute on Aging during the conduct of the study; grants from Alector and personal fees from AVROBIO, Biogen, and ACTC, outside the submitted work.

## Supporting information


**Figure S1**. Mouse Hemibrain and Post mortem Brain Slice Weights. A, A total of three mice (circled in red) were excluded from further analysis for being low outliers (greater than 2 standard deviations below the mean) for hemibrain weight. B, Among the 2‐ to 3‐month‐old mice, *Grn^–/–^* hemibrains were slightly heavier than wild‐type (B, around 12 mg heavier on average, ANOVA effect of genotype, *P* = 0.0015, * = *P* = 0.0445 by Dunnett’s post hoc test). C, However, there was no genotype difference in hemibrain weight among the 12‐ to 13‐month‐old mice (ANOVA effect of genotype, *P* = 0.8965). D, The weight of post mortem tissue slices used for exosome isolation also differed between controls and FTD‐*GRN* patients (*t*‐test, *P* = 0.0325). E, However, post mortem slice weight did not significantly correlate with uncorrected levels of CD81 in fraction 2 (Pearson correlation, r = 0.4077, r^2^ = 0.1662, *P* = 0.0931), suggesting that slice weight was not the primary factor driving group differences in exosome levels.

**Figure S2**. Normal Levels of Brain Exosomes in 2‐ to 3‐month‐old *Grn^–/–^* Mice. A, B, Unlike older, *Grn^–/–^* mice, brains from 2‐ to 3‐month‐old *Grn^–/–^* mice did not contain more exosomes than wild‐type littermates based on nanoparticle tracking analysis (A, RM ANOVA effect of genotype, *P* = 0.4153, genotype x particle size interaction, *P* = 0.9983, B, ANOVA effect of genotype, *P* = 0.6413). C, Fraction 2 from brains of 2‐ to 3‐month‐old *Grn^–/–^* mice did not contain more protein than wild‐type littermates (ANOVA effect of genotype, *P* = 0.0053, but Dunnett’s post hoc test, *P* = 0.1556). D–F, Levels of exosomal marker proteins also did not differ between 2‐ to 3‐month‐old *Grn^–/–^* and wild‐type littermates (d, CD81, ANOVA effect of genotype, *P* = 0.9458, E, Flotillin‐1, ANOVA effect of genotype, *P* = 0.3296, f, HSP‐70, ANOVA effect of genotype, *P* = 0.3643). All data are corrected for hemibrain weight except for the nanoparticle tracking profiles in a. n = 12–14 mice per genotype.

**Figure S3**. GluR4 is not Elevated in Brain Homogenates from 12‐ to 13‐month‐old *Grn^–/–^* Mice. Immunoblot of brain homogenates (Fraction H from Figure 1A) of 12‐ to 13‐month‐old wild‐type and *Grn^–/–^* mice did not reveal an elevation in total brain GluR4 as was observed in the exosomal fraction (Figure 3D,E). Instead, *Grn^–/–^* mice trended toward having less GluR4 than wild‐type mice (*t*‐test, *P* = 0.0738). n = 7 wild‐type and 6 *Grn^–/–^* mice

**Figure S4**. Elevated CD9 and Flotillin‐1 in a *GRN* Mutation Carrier Who Subsequently Developed MCI. A clinically normal *GRN* mutation carrier who went on to develop amnestic MCI (Table 2, case 23, circled in red above) had the highest levels of CD9 (A) and flotillin‐1 (B) among the presymptomatic *GRN* carriers in this study. Data are reproduced from Figure 5E and F with symptomatic *GRN* patients excluded to better visualize the distribution of controls and presymptomatic *GRN* carriers.

**Table S1**. Normalized Spectra and Unique Peptide Counts from Mouse Brain EVs.
