# Supplementary material for: Elevated levels of extracellular vesicles in progranulin‐deficient mice and FTD‐GRN Patients
Source: Ann Clin Transl Neurol. 2020 Nov 16;7(12):2433–49. doi: 10.1002/acn3.51242 (PMC7732244; doi:10.1002/acn3.51242)
Supplement: Supplementary file 1 — Figure S1. Mouse Hemibrain and Post mortem Brain Slice Weights. A, A total of three mice (circled in red) were excluded from further analysis for being low outliers (greater than 2 standard deviations below the mean) for hemibrain weight. B, Among the 2‐ to 3‐month‐old mice, Grn–/– hemibrains were slightly heavier than wild‐type (B, around 12 mg heavier on average, ANOVA effect of genotype, P = 0.0015, * = P = 0.0445 by Dunnett’s post hoc test). C, However, there was no genotype difference in hemibrain weight among the 12‐ to 13‐month‐old mice (ANOVA effect of genotype, P = 0.8965). D, The weight of post mortem tissue slices used for exosome isolation also differed between controls and FTD‐GRN patients (t‐test, P = 0.0325). E, However, post mortem slice weight did not significantly correlate with uncorrected levels of CD81 in fraction 2 (Pearson correlation, r = 0.4077, r2 = 0.1662, P = 0.0931), suggesting that slice weight was not the primary factor driving group differences in exosome levels. [file ACN3-7-2433-s001.pdf]

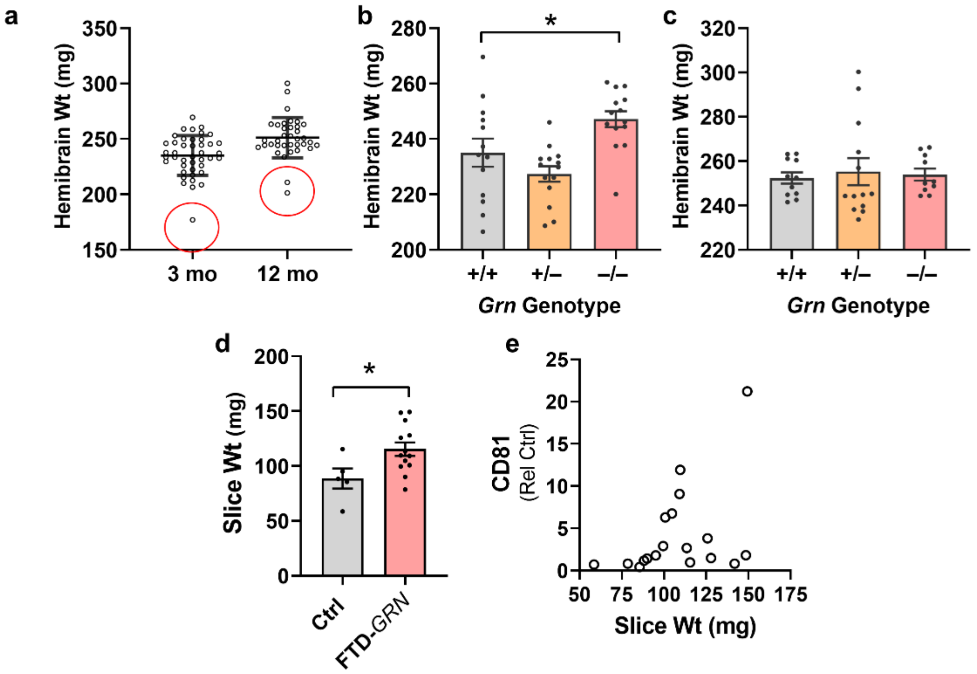

Figure S1 – Mouse Hemibrain and Post-mortem Brain Slice Weights.

a, A total of three mice (circled in red) were excluded from further analysis for being low outliers (greater than 2 standard deviations below the mean) for hemibrain weight. **b,** Among the 2–3 month-old mice, *Grn*$^{-/-}$ hemibrains were slightly heavier than wild-type (**b**, around 12 mg heavier on average, ANOVA effect of genotype, p = 0.0015, * = p = 0.0445 by Dunnett's post-hoc test). **c**, However, there was no genotype difference in hemibrain weight among the 12–13 month-old mice (ANOVA effect of genotype, p = 0.8965). **d**, The weight of post-mortem tissue slices used for EV isolation also differed between controls and FTD-*GRN* patients (*t*-test, p = 0.0325). **e,** However, post-mortem slice weight did not significantly correlate with uncorrected levels of CD81 in fraction 2 (Pearson correlation, r = 0.4077, r^2 = 0.1662, p = 0.0931), suggesting that slice weight was not the primary factor driving group differences in EV levels.
