# Supplementary material for: Elevated levels of extracellular vesicles in progranulin‐deficient mice and FTD‐GRN Patients
Source: Ann Clin Transl Neurol. 2020 Nov 16;7(12):2433–49. doi: 10.1002/acn3.51242 (PMC7732244; doi:10.1002/acn3.51242)
Supplement: Supplementary file 2 — Figure S2. Normal Levels of Brain Exosomes in 2‐ to 3‐month‐old Grn–/– Mice. A, B, Unlike older, Grn–/– mice, brains from 2‐ to 3‐month‐old Grn–/– mice did not contain more exosomes than wild‐type littermates based on nanoparticle tracking analysis (A, RM ANOVA effect of genotype, P = 0.4153, genotype x particle size interaction, P = 0.9983, B, ANOVA effect of genotype, P = 0.6413). C, Fraction 2 from brains of 2‐ to 3‐month‐old Grn–/– mice did not contain more protein than wild‐type littermates (ANOVA effect of genotype, P = 0.0053, but Dunnett’s post hoc test, P = 0.1556). D–F, Levels of exosomal marker proteins also did not differ between 2‐ to 3‐month‐old Grn–/– and wild‐type littermates (d, CD81, ANOVA effect of genotype, P = 0.9458, E, Flotillin‐1, ANOVA effect of genotype, P = 0.3296, f, HSP‐70, ANOVA effect of genotype, P = 0.3643). All data are corrected for hemibrain weight except for the nanoparticle tracking profiles in a. n = 12–14 mice per genotype. [file ACN3-7-2433-s002.pdf]

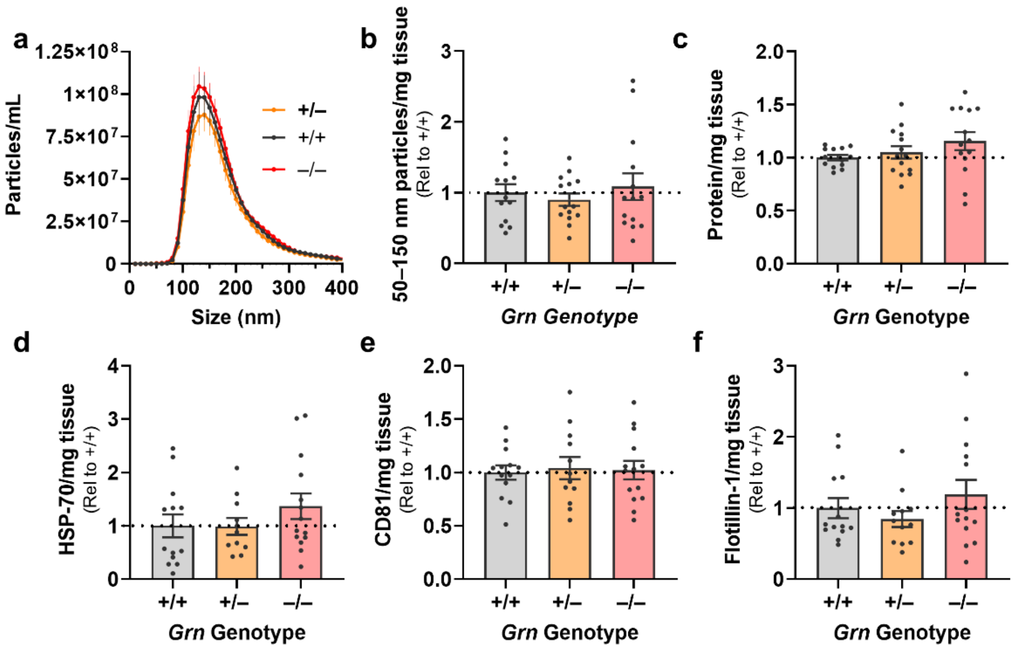

Figure S2 – Normal Levels of Brain EVs in 2–3-month-old *Grn*$^{-/-}$ Mice

a, b, Unlike older, *Grn*$^{-/-}$ mice, brains from 2–3-month-old *Grn*$^{-/-}$ mice did not contain more EVs than wild-type littermates based on nanoparticle tracking analysis (**a**, RM ANOVA effect of genotype, p = 0.4153, genotype x particle size interaction, p = 0.9983, **b**, ANOVA effect of genotype, p = 0.6413). **c**, Fraction 2 from brains of 2–3-month-old *Grn*$^{-/-}$ mice did not contain more protein than wild-type littermates (ANOVA effect of genotype, p = 0.0053, but Dunnett's post-hoc test, p = 0.1556). **d–f**, Levels of EV marker proteins also did not differ between 2–3-month-old *Grn*$^{-/-}$ and wild-type littermates (**d**, HSP-70, ANOVA effect of genotype, p = 0.3643, **e**, CD81, ANOVA effect of genotype, p = 0.9458, **f**, Flotillin-1, ANOVA effect of genotype, p = 0.3296). All data are corrected for hemibrain weight except for the nanoparticle tracking profiles in **a**. n = 12–14 mice per genotype.
