# Supplementary material for: Elevated levels of extracellular vesicles in progranulin‐deficient mice and FTD‐GRN Patients
Source: Ann Clin Transl Neurol. 2020 Nov 16;7(12):2433–49. doi: 10.1002/acn3.51242 (PMC7732244; doi:10.1002/acn3.51242)
Supplement: Supplementary file 3 — Figure S3. GluR4 is not Elevated in Brain Homogenates from 12‐ to 13‐month‐old Grn–/– Mice. Immunoblot of brain homogenates (Fraction H from Figure 1A) of 12‐ to 13‐month‐old wild‐type and Grn–/– mice did not reveal an elevation in total brain GluR4 as was observed in the exosomal fraction (Figure 3D,E). Instead, Grn–/– mice trended toward having less GluR4 than wild‐type mice (t‐test, P = 0.0738). n = 7 wild‐type and 6 Grn–/– mice [file ACN3-7-2433-s003.pdf]

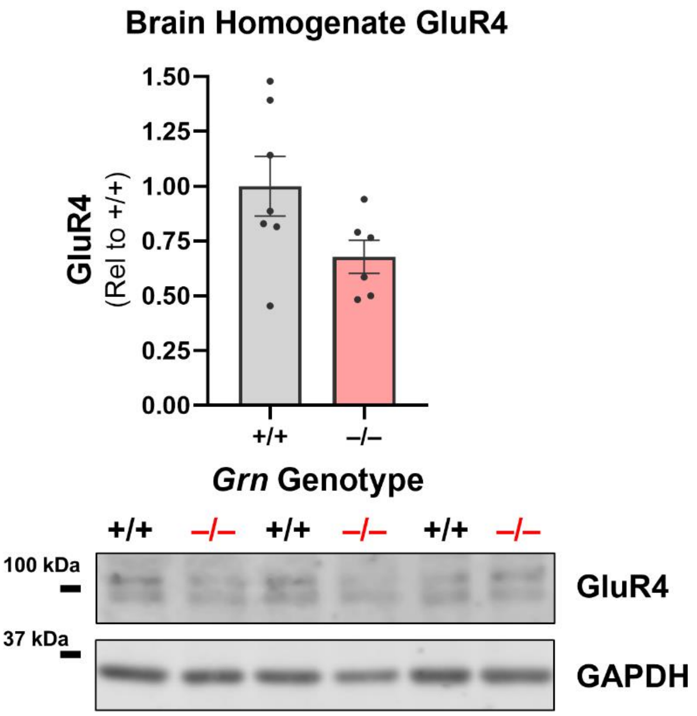

Figure S3 – GluR4 is not Elevated in Brain Homogenates from 12–13-month-old *Grn*$^{-/-}$ Mice.

Immunoblot of brain homogenates (Fraction H from Fig. 1a) of 12–13-month-old wild-type and *Grn*$^{-/-}$ mice did not reveal an elevation in total brain GluR4 as was observed in the EV fraction (Fig. 3d,e). Instead, *Grn*$^{-/-}$ mice trended toward having less GluR4 than wild-type mice (*t*-test, $p = 0.0738$). n = 7 wild-type and 6 *Grn*$^{-/-}$ mice.
