# Supplementary material for: Elevated levels of extracellular vesicles in progranulin‐deficient mice and FTD‐GRN Patients
Source: Ann Clin Transl Neurol. 2020 Nov 16;7(12):2433–49. doi: 10.1002/acn3.51242 (PMC7732244; doi:10.1002/acn3.51242)
Supplement: Supplementary file 4 — Figure S4. Elevated CD9 and Flotillin‐1 in a GRN Mutation Carrier Who Subsequently Developed MCI. A clinically normal GRN mutation carrier who went on to develop amnestic MCI (Table 2, case 23, circled in red above) had the highest levels of CD9 (A) and flotillin‐1 (B) among the presymptomatic GRN carriers in this study. Data are reproduced from Figure 5E and F with symptomatic GRN patients excluded to better visualize the distribution of controls and presymptomatic GRN carriers. [file ACN3-7-2433-s004.pdf]

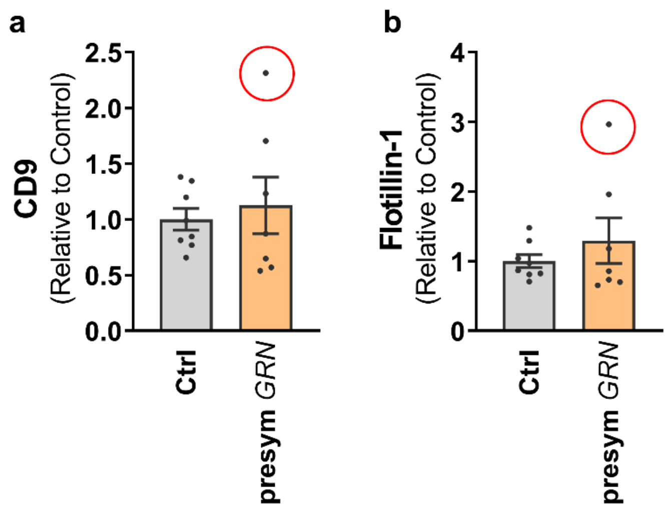

Figure S4 – Elevated CD9 and Flotillin-1 in a *GRN* Mutation Carrier Who Subsequently Developed MCI.

The plasma EV pellet from a clinically normal *GRN* mutation carrier who went on to develop amnestic MCI (Table 2, case 23, circled in red above) had the highest levels of CD9 (**a**) and flotillin-1 (**b**) among the presymptomatic *GRN* carriers in this study. Data are reproduced from Fig. 5e and f with symptomatic *GRN* patients excluded to better visualize the distribution of controls and presymptomatic *GRN* carriers.
